# Dual-Probe Near-Field Phaseless Antenna Measurement System on Board a UAV

**DOI:** 10.3390/s19214663

**Published:** 2019-10-27

**Authors:** María García Fernández, Yuri Álvarez López, Fernando Las-Heras

**Affiliations:** Area of Signal Theory and Communications, University of Oviedo, 33203 Gijón, Spain; garciafmaria@uniovi.es (M.G.F.); flasheras@uniovi.es (F.L.-H.)

**Keywords:** on-site antenna measurement, dual probes, high directive antennas, dual-band Real Time Kinematics (RTK), phaseless techniques, Unmanned Aerial Vehicles (UAVs), antenna diagnostics

## Abstract

On-site antenna measurement has been recently attracting an increasing interest in order to assess the antenna performance in real operational environments. The complexity and cost of these kind of measurements have been significantly cut down due to recent developments in unmanned aerial vehicles’ (UAVs) hardware and antenna measurement post-processing techniques. In particular, the introduction of positioning and geo-referring subsystems capable of providing centimeter-level accuracy together with the use of phase retrieval techniques and near-field to far-field transformation algorithms, have enabled near field measurements using UAVs. This contribution presents an improved UAV-based on-site antenna measurement system. On the one hand, the simultaneous acquisition on two measurement surfaces has been introduced and calibrated properly, thus reducing geo-referring uncertainties and flight time. On the other hand, the positioning and geo-referring subsystem has been enhanced by means of a dual-band real time kinematics (RTK) unit. The system capabilities were validated by measuring an offset reflector antenna, and the results were compared with the measurements at the spherical range in the anechoic chamber and with the measurements collected with a previous version of the implemented system.

## 1. Introduction

In the framework of novel wireless communications systems, such as 5G or Internet of Things (IoT), unmanned aerial vehicles (UAVs) are playing an important role for network deployment and monitoring. For example, UAVs are being introduced as network nodes for data gathering in large areas [1,2] and to extend mobile networks coverage by means of drone base stations [3].

Within this framework, on-site calibration [4] and measurement [5,6,7,8,9,10] of antennas for wireless communications have gained interest due to the advances in UAVs technology, which simplify the procedures for on-site measurements. Antenna measurement in anechoic chambers provide an accurate characterization of the antenna parameters (e.g., radiation pattern, gain, return losses). However, it must be taken into account that these parameters can be affected by the environment surrounding the place where the antenna is deployed. For example, buildings and obstacles could result in multipath contributions that could modify the coverage. On-site antenna measurements allow the assessment of the radiation performance of the antenna considering the influence of the surrounding environment.

The improvements in geo-referring and positioning subsystems on board the UAVs, capable of providing centimeter-level accuracy, have also enabled on-site antenna measurement at higher frequency bands. The current state-of-the-art on on-site antenna measurement has shown the feasibility of these systems up to C-band [5,6].

The most common geo-referring and positioning subsystems that can be mounted on UAVs are global navigation satellite systems (GNSS), real time kinematics (RTK), inertial measurement units (IMUs), laser rangefinders, photogrammetry systems and barometers. A combination of these systems can lead to geo-referring accuracies within a few centimeters. Ground-based laser trackers can also be used to increase accuracy by one order of magnitude [6].

Most of the prototypes and systems for on-site antenna measurement using UAVs are intended to operate in the far field (FF) region of the antenna-under-test (AUT) which, in the case of electrically large antennas (e.g., reflector antennas, mobile networks base station antennas) can be hundreds of meters away from the AUT [7,8]. As explained in [9], there are some restrictions for the direct measurement of the FF pattern using UAVs, namely safety restrictions (forbidden flight areas) and UAV flight autonomy. These issues can be overcome if the measurements are carried out in the near field (NF) region of the AUT, so that the furthest distance from the AUT to the UAV is not greater than 10–20 m. The feasibility of NF measurements using UAV-based systems has been proved in [9,10,11,12]. 

In order to recover the FF pattern of the AUT from NF measurements, near field-to-far field (NF-FF) transformation techniques must be applied [13]. For this reason, complex (i.e., amplitude and phase) NF measurements are required. However, complex NF measurements require more expensive (and weighty) hardware to be placed on board the UAV. Besides, phase measurements are more sensitive to geo-referring uncertainties. An alternative to the use of complex receivers is provided by phase retrieval techniques, capable of recovering phase information from amplitude-only measurements [14,15,16,17,18,19]. Once the phase of the field is retrieved, NF-FF transformation techniques can be applied to calculate the AUT radiation pattern. However, NF measurements can be post-processed for antenna diagnostics (e.g., detection of malfunctioning elements).

There are two main groups of phase retrieval methods. On the one hand, holographic techniques have been proved to be very accurate and efficient in terms of measurement time, as just one measurement surface has to be scanned. However, these techniques require an additional hardware component (e.g., a reference antenna or an injected reference signal) [14]. On the other hand, iterative techniques do not need additional hardware elements, but the NF radiated by the AUT must be measured on two or more acquisition surfaces [15,16,17,18]. Thus, for on-site NF antenna measurements using UAVs, the latter group of methods is more suitable, allowing a faster and easier deployment of the system. Nevertheless, recent developments in phaseless antenna measurement techniques using a single acquisition surface [19] are a promising solution aiming to avoid the need of two acquisition surfaces.

In [9,11,20], an UAV-based system for on-site antenna measurement was presented, assessing the performance of the implemented system for antenna diagnostics and characterization. This contribution presents several novelties to improve the measurement accuracy of the system, thus enabling the characterization of high directive antennas, such as reflectors. These improvements are (i) the use of a dual-channel receiver, that allows placing two receiving antennas (probe antennas) at two different distances onboard the UAV, thus collecting NF measurements at two different distances simultaneously; (ii) the introduction of a GNSS dual-band RTK receiver, which increases positioning and geo-referring accuracy and reliability. The former also helps to reduce the flight time, since only one flight is required to gather measurements at two different surfaces. The later contributes to reduce the deployment time, since the convergence time (i.e., the time until a corrected position is obtained) improves compared to a single-band RTK receiver. However, several technical flaws found in the previous version of the prototype for on-site measurements that had an impact in the accuracy of the measurements have been assessed and solved.

The experimental validation was conducted measuring a C-band reflector antenna which, to the authors’ knowledge, has not been carried out before using other state-of-the-art UAV-based antenna measurement systems.

## 2. System Description

### 2.1. Hardware Architecture

**Figure 1:**
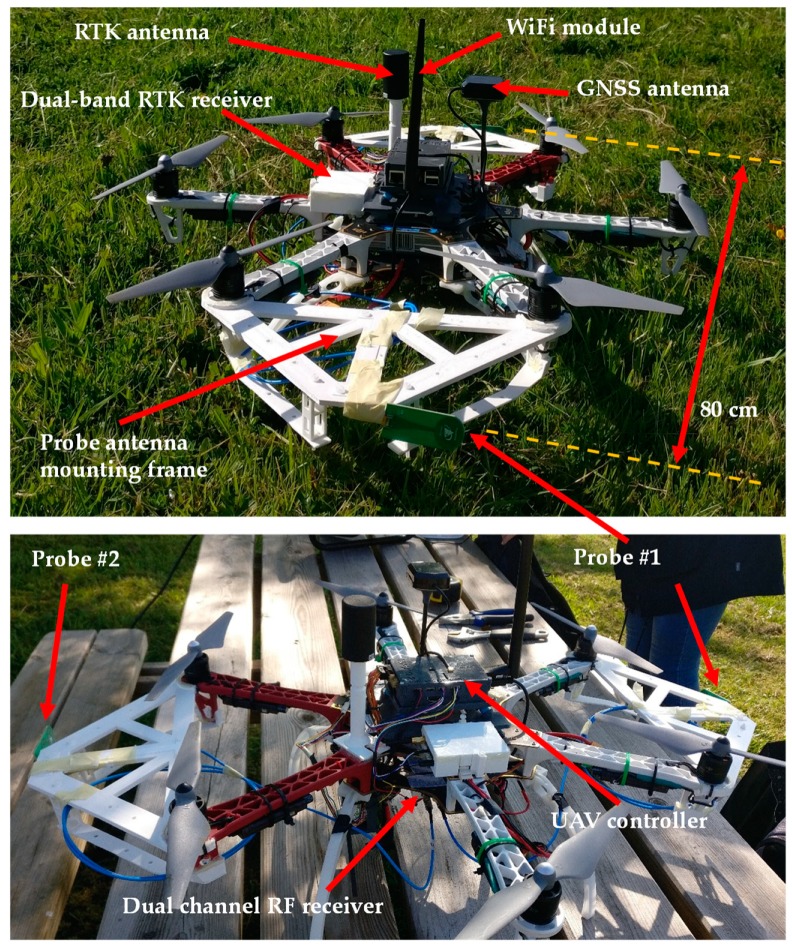
Picture of the implemented prototype, highlighting the placement of the two-probe antenna, the mounting frames for the probe antennas, and the distance between them.

A picture of the implemented UAV-based system for the on-site antenna measurement is shown in Figure 1. The main subsystems onboard the UAV are:

- *Communications subsystem:* Consisting of a wireless local area network (WLAN) operating in the 2.4–2.5 GHz and 5.7–5.8 GHz frequency bands. The radio transmitter and receiver for radiofrequency control (R/C) of the UAV operate at 433 MHz.
- *Control subsystem:* Composed of the UAV controller, which gathers positioning data and radiofrequency measurements, and forwards them to the ground control station that processes geo-referred NF measurements.
- *Radiofrequency subsystem:* Composed of two commercial monopole antennas working in the 4 to 7 GHz frequency band [21] acting as probe antennas for NF measurements, connected to a dual-channel radiofrequency power detector based on the ADL5519 chip [22]. To maximize the distance between the two measurement acquisition surfaces, these probe antennas are placed 80 cm away, as shown in Figure 1. The output of these channels is converted into a digital sequence and sent to the ground station (a laptop) using the WLAN.
- *Positioning and geo-referring subsystem:* Composed of the GNSS-RTK unit onboard the UAV [23]. A laser rangefinder is also integrated to improve height positioning, although the GNSS-RTK unit is accurate enough to avoid the need for a laser rangefinder (it was mandatory for accurate height information in previous versions of the prototype [9,12]). The positioning system is completed by the default positioning components typically included on board UAVs, namely: conventional GNSS receiver, barometer and inertial measurement unit (IMU).

### 2.2. Dual-Channel Receiver Calibration and Mounting

The feasibility of the dual-channel receiver was presented as a proof of concept in [12], where the receiver was placed on board the first version of the developed prototype for on-site antenna measurement. However, it was found that the two input channels were unbalanced, resulting in a 3–4 dB difference in the measured radiofrequency (RF) power, which can be due to small differences in the monopole antenna radiation patterns and the RF cables connecting the antennas and the inputs of the receiver. This unbalance in the dual-channel receiver required the implementation of a calibration stage, conducted as follows:

(1) The UAV is manually placed at a known distance (e.g., 1 m) from an omni-directional transmitting antenna (e.g., a monopole antenna), so that the two monopole antennas onboard the UAV are at the same distance from the transmitting antenna, having the same orientation.
(2) The signal level measured at each channel is recorded and the unbalance between both channels (ΔRF_1,2_(r_1_)) is obtained.
(3) Steps 1) and 2) can be applied for different distances between the UAV and the omni-directional transmitting antenna, yielding ΔRF_1,2_(r_2_), ΔRF_1,2_(r_3_), …, ΔRF_1,2_(r_N_).
(4) Dual-channel unbalance correction factor is estimated as: *mean*{ΔRF_1,2_(r_n_)}, n = 1,2, …, N.

After this calibration stage, the unbalance between the two input channels was reduced to less than 0.5 dB.

To maximize the distance between the two monopoles placed on board the UAV, different solutions were considered. In [12], two plastic tubes were attached to the UAV frame (Figure 1, [12]). However, it was found that they were not robust enough to avoid vibrations induced by the UAV propellers, which have an impact in the measurements geo-referring accuracy. After several in-flight tests, ad-hoc mounting frames were designed to mitigate the vibrations on the monopole antennas. The mounting frames for the monopole antennas, manufactured using 3D printing, are depicted in Figure 1. 

The fact that both the mounting frames for the monopole antennas and the UAV frame are made or plastic reduces scattering and interferences from the UAV itself. Placing the monopole antennas away from the UAV also contributes to mitigate this issue.

### 2.3. GNSS-RTK Unit: Features and Integration in the System

The GNSS-RTK unit on board the UAV [23] is a dual-band multiconstellation GNSS receiver, which is more robust to the multipath and can keep centimeter accuracy even in challenging environments (e.g., with limited sky). However, the convergence time (time required by the GNSS-RTK module to resolve carrier phase ambiguities to integer number, i.e., to achieve a “fix” status) is smaller and it can recover faster from temporary loss of accuracy than single-band GNSS-RTK modules. Therefore, the proposed positioning system provides better accuracy and overall performance compared to the single-band GNSS-RTK unit used in the previous versions of the system [9,12]. Quantitatively, it has been found that the average time to reach a fix status (i.e., to obtain cm-level accuracy coordinates) was reduced from more than 2–3 minutes to less than 10 s. The assessment of the GNSS-RTK systems was done in the same scenario in all the cases (airfield of the University of Oviedo, https://goo.gl/maps/smXzpN1jmTjELpCj6). 

**Figure 2:**
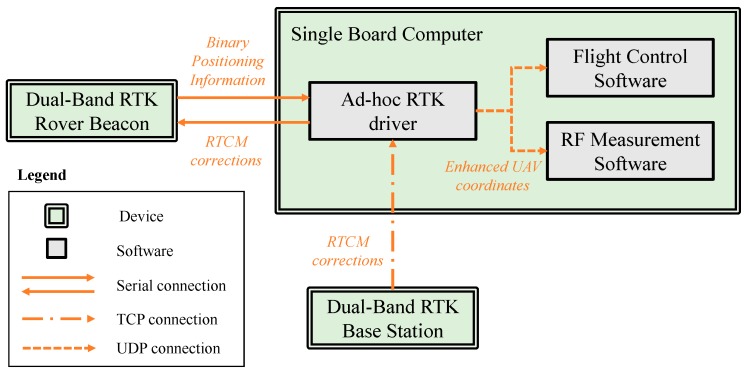
Scheme of the hardware devices associated to the dual-band global navigation satellite systems-real time kinematics (GNSS-RTK) unit, highlighting the software modules that were developed for its integration into the unmanned aerial vehicles (UAV) controller (single board computer).

Concerning the integration of the GNSS-RTK unit [23], a customized dual-band RTK receiver was integrated into the UAV. an ad-hoc driver for the RTK system that runs on a single board computer (SBC) on board the UAV was developed. This driver is in charge of (i) receiving Radio Technical Commission for Maritime Services (RTCM) corrections via the internet using a transmission control protocol (TCP) from an RTK base station (at a fixed position on the ground); (ii) forwarding the RTCM corrections to the RTK rover beacon (on board the UAV); (iii) receiving binary positioning information from the RTK rover beacon and transforming it into human-readable data (obtaining the enhanced coordinates); (iv) forwarding the enhanced coordinates to the flight control software and to the RF measurement software using a user datagram protocol (UDP) connection. A scheme of how these hardware devices and software modules are interconnected is depicted in Figure 2.

Furthermore, the accuracy was also improved by a factor of approximately 3 in all directions of space, with the current dual-band system having an accuracy of 0.5 cm in the horizontal plane and 1 cm in the vertical direction. It must also be noticed that its ambiguity resolution is more robust so it is less likely to lose the fix status. 

As a result, these improvements enable a better UAV navigation and measurement geo-referring accuracies. The former helps to reduce the deviation of the UAV flight path from the desired path defined with waypoints. The latter contributes to improve the accuracy of the antenna diagnostics information and the radiation pattern. 

In the configuration presented in this contribution, the GNSS corrections were taken from a fixed based station of the Spanish Instituto Geodésico Nacional [24]. This allows the deployment of a fixed RTK beacon in the ground (to act as base station for the RTK system) to be avoided, which results in less hardware complexity and faster deployment of the system. The connection to the fixed base station is done by providing 3G/4G connectivity to the WLAN. 

### 2.4. Near Field Measurements Processing

As explained in Section 1, the system proposed in [9,11,20] makes use of the NF measurement data collected on two or more surfaces surrounding the AUT. These measurements are post-processed by means of phase retrieval techniques to recover phase information.

The iterative phase retrieval methods, such as the ones presented in [15,16,17,18], require the NF measured on the two acquisition surfaces to have different spatial distribution, taking advantage of the fact that the field distribution in the NF region depends on the distance to the AUT [13].

The UAV-based systems for on-site antenna measurements are limited by the life of the batteries that feed the systems on board the UAV. The use of a dual-channel radiofrequency power detector connected to two probes mounted on different positions on the UAV enables the simultaneous acquisition of the NF on two different surfaces. Furthermore, geo-referring uncertainties have the same impact in both acquisition surfaces, avoiding cumulative errors if the measurements on each acquisition surface were conducted in two different flights. The latter would increase the measurement time and thus, the operational cost.

As shown in Figure 1, the two 3D-printed plastic structures designed and manufactured to mount the two probe antennas on the UAV frame achieve a maximum separation between the probes of 80 cm. The probes and the two channels of the power detector were tested on the ground to ensure that they exhibited the same performance in terms of dynamic range and sensitivity. However, the calibration procedure explained in Section 2.1 was conducted to correct the unbalance between the channels of the power detector.

The NF measurements acquired on the two measurement surfaces were post-processed using the phaseless sources reconstruction method (pSRM) [16]. This method is based on minimizing a cost function (*F*) relating the measured NF samples on each acquisition surface (*E*_NF,p1_, *E*_NF,p2_) to the NF radiated by an equivalent currents distribution (*M*_eq_) that characterizes the AUT. The pSRM iterates until finding an equivalent currents distribution that radiates the same NF as the measured one. 

**Figure 3:**
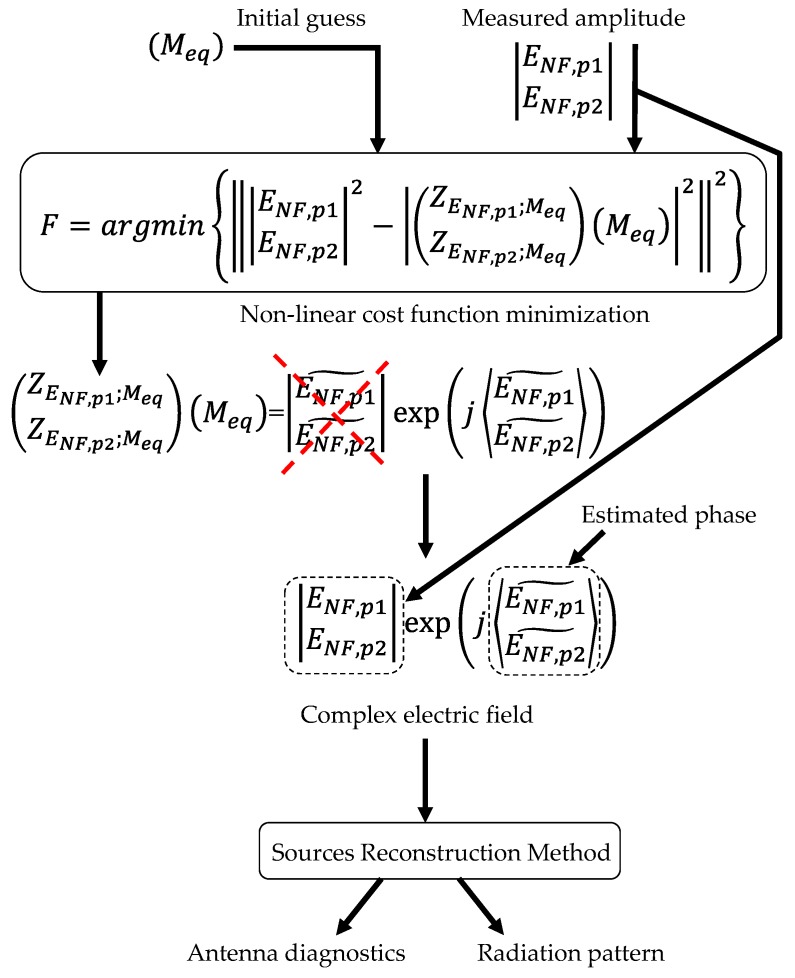
Flowchart of the phaseless sources reconstruction method (pSRM) based on the minimization of a non-linear cost function [12,18].

Concerning the cost function to be minimized, there are two main approaches. On the one hand, the forward-backward implementation of the pSRM is based on the minimization of a linear cost function, which requires working with the complex representation of the NF. Thus, an initial guess of the phase distribution on the measurement surfaces is required. A description of the forward-backward implementation technique is presented in [16] and revisited in [18] in order to assess its performance for on-site antenna measurement. On the other hand, a non-linear cost function relating the amplitude of the measured NF (|*E*_NF,p1_|,|*E*_NF,p2_|) and the amplitude of the NF radiated by the equivalent currents can be set, as illustrated in Figure 3. In this case, the initial guess is done on the equivalent currents, which is simpler than estimating an initial guess for the phase of the NF in the measurement surfaces. The better performance of the pSRM based on non-linear cost function minimization over the forward-backward technique has been analyzed and discussed in [18].

Once convergence is achieved, or the maximum number of cost function minimization iterations has been reached, the output is an equivalent currents distribution that is used to compute the phase of the NF on the measurement surfaces. Then, the complex NF can be used for antenna diagnostics (NF-NF transformation) or for the calculation of the radiation pattern of the AUT (NF-FF transformation).

The reason why pSRM [16,18] was chosen over other iterative phase retrieval techniques [15,17] is due to its capacity to work with non-canonical, non-uniformly sampled acquisition surfaces, which is likely to occur in the case of airborne-based measurement systems. As explained in [16], either the SRM or its phaseless version, pSRM, are based on the electromagnetic integral equations relating an equivalent currents distribution defined on a surface enclosing the AUT with the field radiated by these equivalent currents. These integral equations are discretized using piecewise basis functions. Other phaseless techniques for antenna measurement are based on wave mode expansion (e.g., planar [17], cylindrical [25], and spherical [26]), which are computationally much more efficient than the SRM/pSRM, but they require the NF to be uniformly sampled. Compressed sensing techniques can deal with sparse measurements as well [27], even in the case of phaseless measurements [28], resulting in measurement time reduction. However, sparse sampling patterns can be complex to be translated into a UAV pre-defined flight path. 

## 3. Experimental Validation

**Figure 4:**
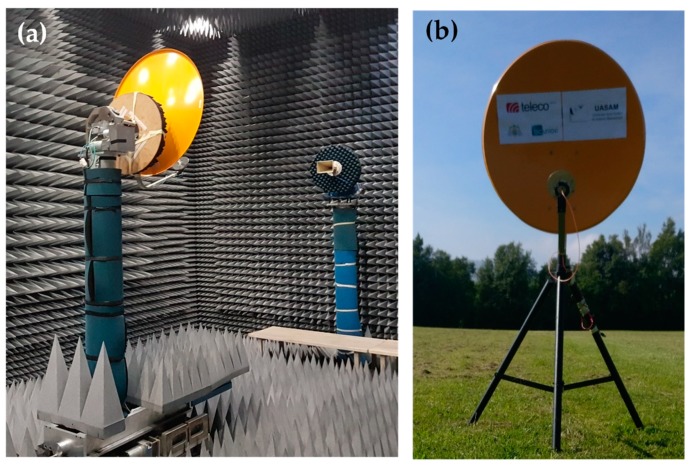
Pictures of the reflector antenna chosen as AUT. (**a**) Measurement at spherical range in anechoic chamber of the University of Oviedo; (**b**) Placed outdoors for on-site measurement.

The aforementioned improvements in the proposed UAV-based system for on-site antenna measurements have been assessed experimentally. For this purpose, an offset reflector antenna working at 4.65 GHz, and fed with a circularly-polarized helix antenna, was selected as AUT (Figure 4). This AUT has a directivity of ~30 dB, being the main beam (which has a −10 dB beamwidth of BW_−10dB_ ~12°) tilted *θ* = 20° with respect to the vertical axis for this scenario. For this AUT, the FF distance is approximately 8 m (124 λ) at the frequency of 4.65 GHz. One of the reasons that led to the choice of this antenna was to prove the capabilities of the system for testing high directive antennas, which have been widely used in applications, such as radioastronomy or satellite communications. For reference purposes, the AUT was also measured at the spherical range in anechoic chamber of the University of Oviedo (Figure 4a). As this AUT was chosen for the on-site measurements conducted with the previous version of the prototype presented in [12], the results shown in this contribution are compared with the results in [12] to highlight how the proposed improvements have an impact in the measurement results.

**Figure 5:**
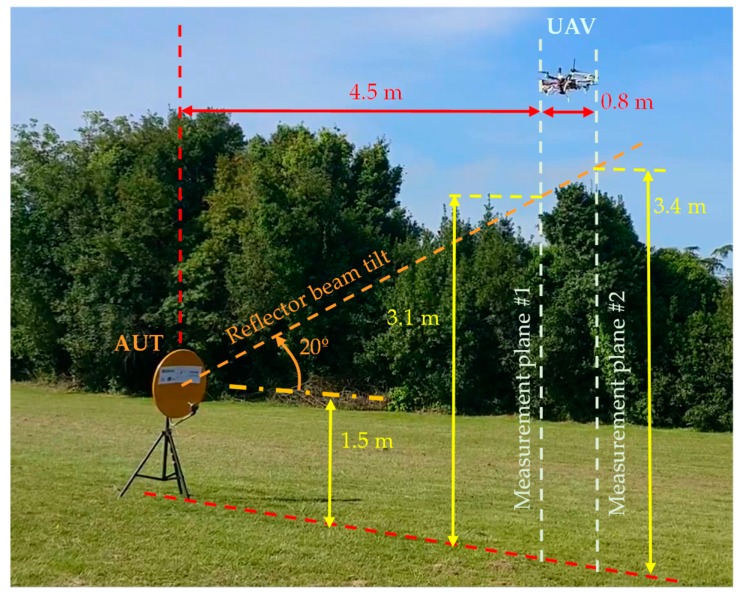
Picture of the UAV and the reflector antenna placed on top of a 1.5 m high metallic mast, and the scheme of the distances of the measurement domain.

As shown in Figure 4b and Figure 5, the AUT was mounted on top of a 1.5 m height metallic mast, and then placed in the airfield of the University of Oviedo. The transmitted signal was a tone at 4.65 GHz generated using an oscillator and later amplified up to 15 dBm.

First, a preliminary flight along a vertical axis 4.5 m (70 λ) away from the AUT was conducted to determine the placement of the maximum of the AUT main beam. Once its position was found, the flight path and, consequently, the acquisition surfaces, were defined as shown in Figure 5. 

**Figure 6:**
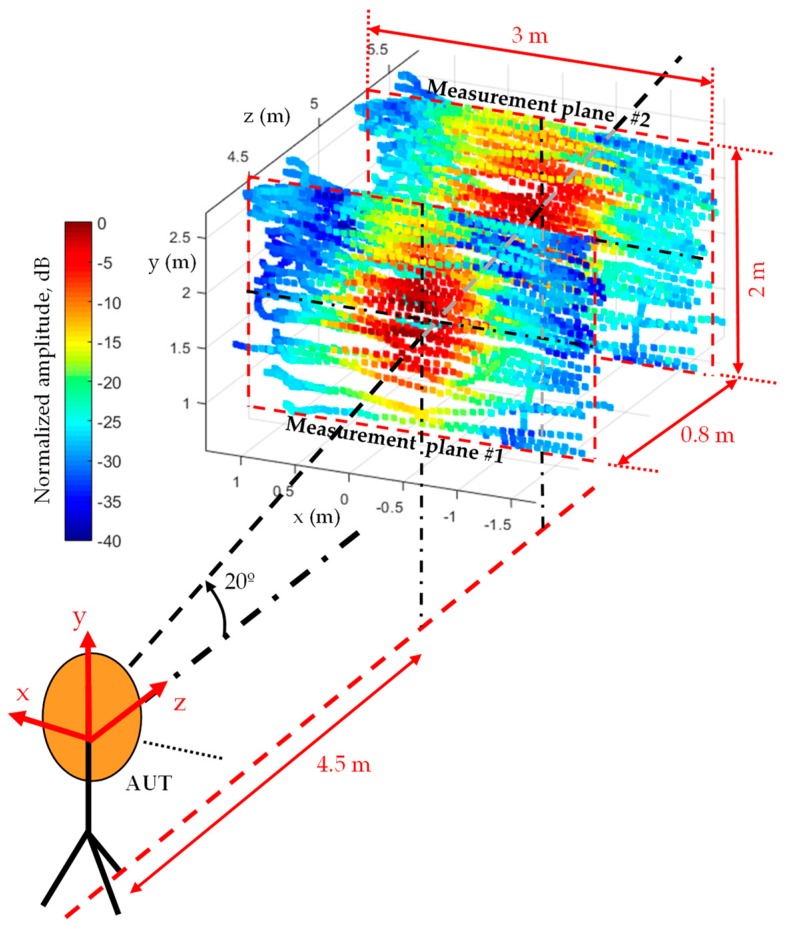
Amplitude (normalized, in dB) of the near field (NF) measured at the geo-referred UAV flight path positions. Notice the offset in the vertical axis (y-axis) of the acquisition surfaces in order to capture the tilted main beam of the reflector antenna. The origin of the coordinates system is at the antenna.

The implemented system allows defining the different kind of measurement domains, e.g., planar, cylindrical, spherical [29]. In the case of high directive antennas, such as the AUT selected for validation purposes, a planar measurement domain is sufficient for the proper characterization of the AUT, since most of the power radiated is expected to be within the acquisition domain. An acquisition domain of (*x*, *y*) = (4, 2.4) m size was defined, later truncated to (*x*, *y*) = (3, 2) m to reduce the amount of NF data, as shown in Figure 6. 

**Figure 7:**
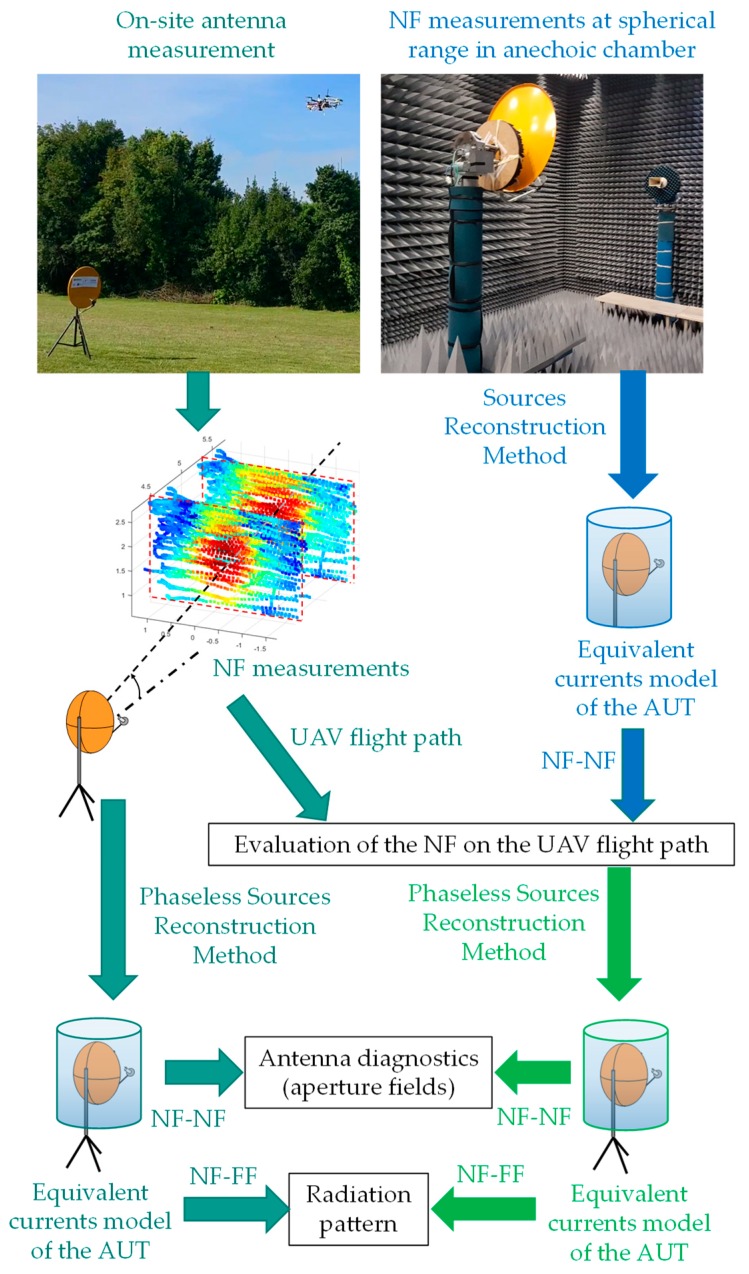
Flowchart of the validation methodology followed in the example presented in Section 3.

The convergence of the iterative phase retrieval methods, such as the pSRM, is influenced by the distance between the acquisition surfaces and the AUT, as well as the spacing between the acquisition surfaces. A quantitative assessment of the influence of these parameters was provided for this AUT, taking advantage of the fact that this antenna was characterized at the spherical range in the anechoic chamber. In particular, an analysis based on simulated flight path positions using the NF calculated on these positions from an electromagnetic model of the AUT was conducted. This model was obtained from the NF measurements at a spherical range in the anechoic chamber (Figure 7) following the methodology presented in [18].

**Figure 8:**
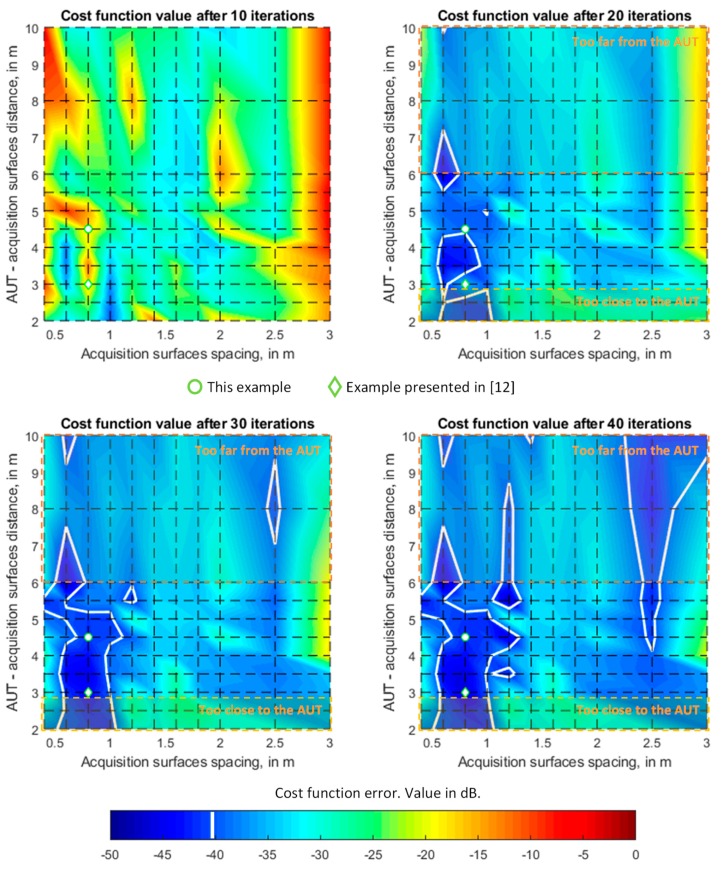
Analysis of the pSRM convergence (cost function error) for different spacing between acquisition surfaces and several distances to the AUT. The simulated geo-referred flight path acquisition points have been considered, evaluating the NF radiated by an electromagnetic model of the AUT at these positions.

The results depicted in Figure 8 show that the best cases correspond to a spacing between acquisition surfaces within 60 cm–120 cm (9.3 λ–18.6 λ) and an AUT-acquisition surface distance not greater than 5 m (77.5 λ). However, the minimum and maximum UAV flight distances from the AUT (depicted in Figure 8) must be taken into account: too close increases the risk of accidental collision (e.g., due to a sudden wind gust) and too far increases the size of the acquisition grid. Based on the results of Figure 8, it was decided to set the AUT-UAV distance at approximately 4.5 m away from the AUT (1.5 m further than in [12]). The acquisition distances were 4.5 (70 λ) for the first measurement grid and 5.3 (82 λ) for the second one (see Figure 5 and Figure 6).

A grid spacing in height (y-axis) of 5 cm (0.77 λ) was chosen as a trade-off between the sampling rate and the number of scans that can be performed taking into account the capacity of the UAV batteries. If the grid spacing was set to 0.5 λ (3.2 cm) to ensure Nyquist sampling rate [13], this would result in 75 scans along *x*-axis, which would require replacing the battery before the acquisition can be completed. With a grid spacing of 5 cm, the number of scans is reduced to 48, avoiding UAV battery replacement in the middle of the measurement process. Nevertheless, in the case of planar NF measurements of directive antennas, the relaxation of the sampling rate above Nyquist can be admitted, provided the grating lobes do not fall within the FF angular margin of validity.

For validation purposes only, the horizontal (x-axis) component of the field was measured, as it can be deduced from the position of the monopole probe antennas in Figure 1. Nevertheless, the vertical (y-axis) component of the NF could be acquired as well by rotating the two probes 90° and repeating the flight using the same flight waypoints defined for the measurement of the horizontal component. 

Once the flight path was defined, the AUT was measured using the UAV-based system described in Section 2. The NF measurements were simultaneously acquired in both the acquisition surfaces after a 12 minutes flight. The overall number of samples collected was 6016, in agreement with the number of samples that would result from a uniform half-a-wavelength (λ/2) sampling of a 3 m × 2 m plane, which would be 5766. A video of the UAV-based system in operation can be watched at: https://youtu.be/k3CleGTkHxE (see Supplementary Materials). 

**Figure 9:**
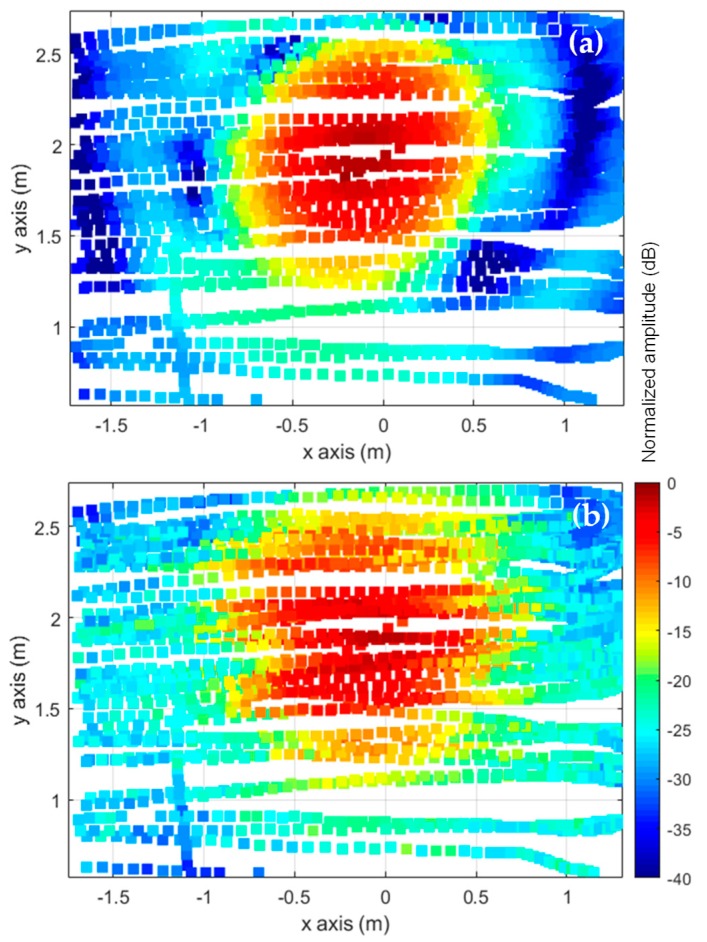
NF on the measurement surface #1 depicted in Figure 6. Comparison between (**a**) the NF estimated from an equivalent currents model of the AUT using anechoic chamber measurements and (**b**) on-site NF measurements.

Regarding validation purposes, on-site NF measurements were compared with the NF estimated from an equivalent currents model of the AUT obtained from complex measurements at a spherical range in the anechoic chamber. The NF radiated by the equivalent currents model was evaluated at the positions shown in Figure 6, as outlined in the flowchart depicted in Figure 7. The comparison of the NF measured and evaluated at the two acquisition surfaces is depicted in Figure 9. Despite the main lobe of the reflector antenna being properly characterized, the positioning and geo-referring uncertainties as well as the limited dynamic range of the dual-channel receiver introduce some distortion in NF measurements acquired with the UAV-based system (Figure 9b).

Next, the amplitude-only information of the NF acquired at the two measurement surfaces shown in Figure 6 was introduced in the pSRM to recover an equivalent currents model of the AUT, as outlined in the flowchart of Figure 3. The recovered equivalent currents model of the AUT is used to calculate the NF on the AUT aperture plane as well as the radiation pattern. For validation purposes, the same procedure followed with the on-site NF measurements was conducted with the NF on the measurement surfaces estimated from the NF measurements at a spherical range in the anechoic chamber (flowchart of Figure 7). Concerning the pSRM convergence, it was achieved after 33 iterations when the NF inputs were on-site measurements, and after 25 iterations when the NF was estimated from the anechoic chamber measurements. The calculation time was 55 s in a conventional laptop, that is, ~8% of the measurement time using the UAV.

**Figure 10:**
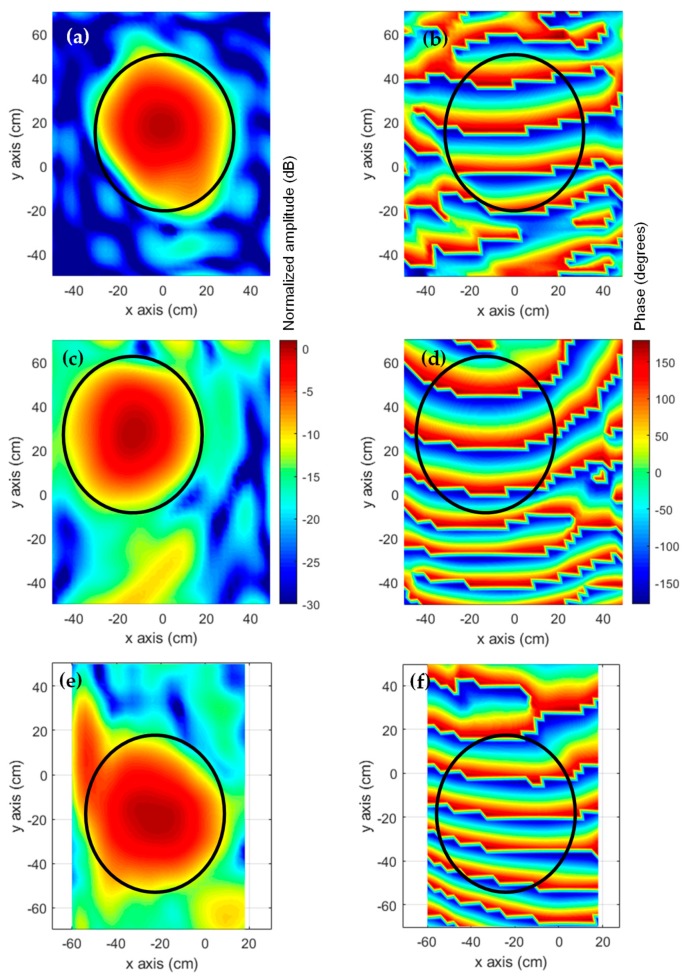
Amplitude (**a**) and phase (**b**) of the reconstructed aperture fields from complex NF measurements at spherical range in anechoic chamber. Amplitude (**c**) and phase (**d**) of the reconstructed aperture fields from on-site NF measurements, current prototype. Amplitude (**e**) and phase (**f**) of the reconstructed aperture fields from on-site NF measurements, previous prototype [12]. Solid black line represents the projected profile of the reflector antenna.

The NF on the AUT aperture plane is depicted in Figure 10. Figure 10a,b correspond to the amplitude and phase distribution of the NF when the anechoic chamber measurements are considered, whereas Figure 10c,d are the estimated aperture fields calculated from the on-site NF measurements. In both cases, the amplitude distribution fits the shape and size of the reflector antenna. In the case of the phase distribution, the typical phase-shift of the off-centered reflector antennas can be noticed along the vertical direction (y-axis). For comparison purposes, the aperture fields distribution from the proof-of-concept shown in [12] are depicted in Figure 10e,f. In this case, the AUT diagnostics is not as accurate as in the improved prototype, as some artifacts appear outside the projected aperture of the reflector antenna (upper left side). The differences in the phase distribution are mainly due to the different tilt of the reflector antenna in this validation example and in [12].

**Figure 11:**
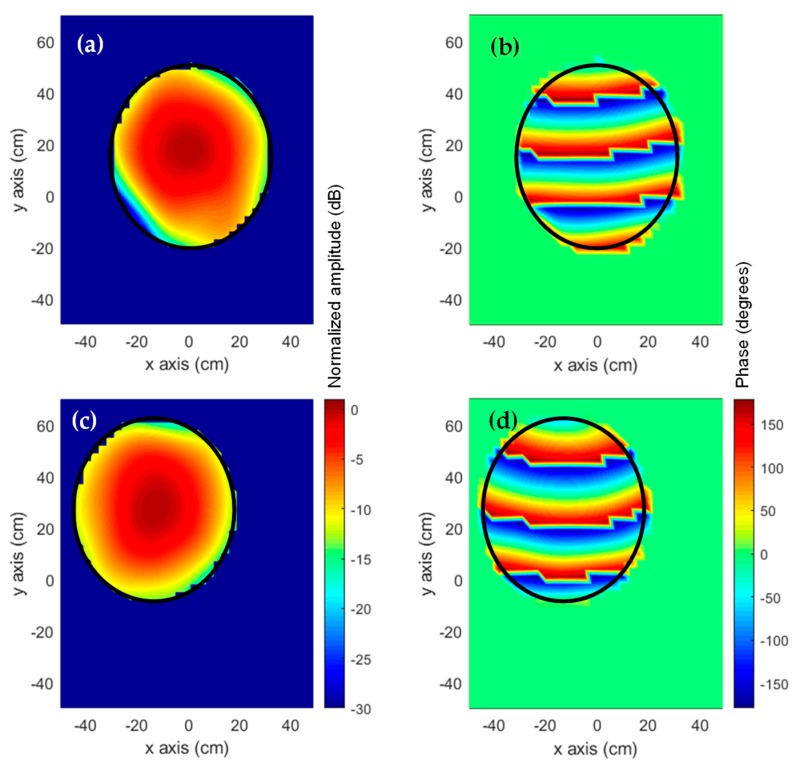
Filtered aperture fields on the AUT aperture plane. Amplitude (**a**) and phase (**b**) of the reconstructed aperture fields from complex NF measurements at spherical range in anechoic chamber. Amplitude (**c**) and phase (**d**) of the reconstructed aperture fields from the on-site NF measurements using amplitude-only information. The solid black line represents the projected profile of the reflector antenna.

**Figure 12:**
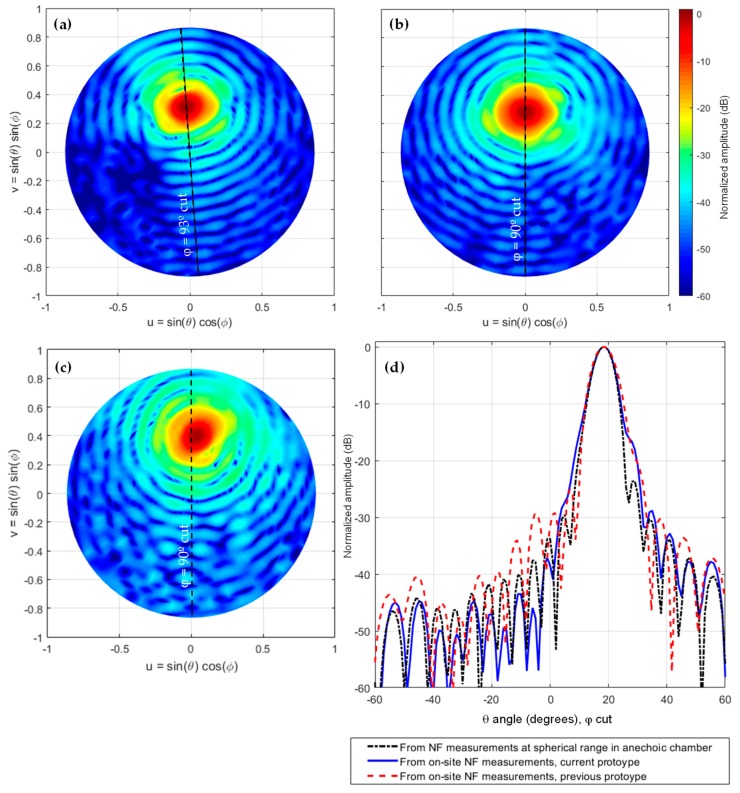
Far field pattern. (**a**) From complex NF measurements at spherical range in anechoic chamber; (**b**) From amplitude-only information from on-site NF measurements, current prototype; (**c**) From amplitude-only information from on-site NF measurements, previous prototype [12]; (**d**) Comparison between NF-FF transformation from measurements at spherical range in anechoic chamber and on-site measurements.

From the aperture fields, the AUT radiation pattern can be calculated by means of NF-FF transformation. To eliminate the noise and artifacts observed in Figure 10 which are outside the area corresponding to the AUT physical aperture size (solid black line in Figure 10), filtering can be applied. The aperture fields outside the AUT physical aperture size are discarded. Thus, the radiation pattern was calculated from the aperture fields of Figure 11. The far field pattern is depicted in Figure 12. It can be observed that the reflector antenna was slightly tilted when measured at the spherical range in the anechoic chamber. For a better comparison, the main beam vertical cut is shown in Figure 12c. The agreement between the far field calculated from the on-site NF-measurements using the UAV-based antenna measurement system, and the one calculated from the NF measurements at the spherical range in the anechoic chamber can be observed.

## 4. Discussion

From the results shown in Figure 12, it can be noticed that the main beam is wider in the case of the on-site NF measurements. The most likely reason is that the reflections on the ground (see Figure 5), due to the realistic environment where the AUT is placed, make the measured NF data different from that of the ideal case of the anechoic environment in chamber measurements, thus resulting in the filling of the sidelobes adjacent to the main beam. It must be remarked that, in particular, the filling of the sidelobe at θ = 25° can be observed using the measurements taken both with the previous and current prototype. This result justifies the importance of the on-site measurements, as they allow assessing the influence of the environment surrounding the AUT.

Concerning the quantitative assessment of the AUT radiation pattern, Table 1 shows a comparison of the radiation pattern parameters, namely −3 dB beamwidth and directivity. In the case of using NF amplitude-only data, the UAV-based system for the antenna measurement presented in this contribution provides similar results as the NF measurements at the spherical range in the anechoic chamber. Furthermore, the improvements in positioning and geo-referring, as well as a better characterization of the dual-channel receiver, result in a better estimation of the radiation pattern with respect to the proof-of-concept shown in [12], as can be concluded from Table 1 and Figure 12.

Another important result from this contribution is the capability of the pSRM for measuring off-centered high-directive antennas, providing diagnostics and radiation characterization. In previous works, such as [30], the validations for this kind of antennas were based on the simulations.

## 5. Conclusions

Several improvements of a UAV-based system for the on-site antenna measurements were presented in this contribution. The use of a dual-channel radiofrequency power detector allows for the simultaneous acquisition of the NF measurements on two measurement surfaces, thus reducing the positioning and geo-referring uncertainties with respect to the case in which two independent flights are needed for the acquisition on each surface. However, the introduction of a dual-band multiconstellation RTK unit improves GNSS-RTK reliability. Both advances also yield smaller flight and deployment times, as well as more accurate measurements compared to previous prototypes of the implemented system. These features would make measurements at higher frequency bands feasible.

The results prove the capability of the improved system for the characterization of high directive antennas, thus broadening the field of application where UAV-based antenna measurement systems can be introduced. 

## 6. Patents

The work presented in this contribution is related to the patent “Airborne System and Method for the characterization and measurement of radiating systems or antennas”. Publication No. WO/2018/158472. International Application No. PCT/ES2018/000015. Priority date: 03/03/2017. International filing date: 02/03/2018. University of Oviedo, University of Vigo. Available online: https://worldwide.espacenet.com/publicationDetails/biblio?CC=WO&NR=2018158472A1&KC=A1&FT=D (accessed on 1 August 2019).

## Figures and Tables

**Table 1:** Radiation pattern parameters of the AUT.

Measurement	Directivity	−3 dB Beamwidth
Anechoic chamber measurements. NF-FF, complex NF.	29.8 dB	6.6°
Anechoic chamber measurements. NF-FF, amplitude-only data.	29.5 dB	6.8°
On-site measurements. Previous prototype [12]. Amplitude-only.	29.4 dB	6.9°
On-site measurements. Current prototype. Amplitude-only.	29.6 dB	6.7°

## Supplementary Materials

The following are available online at https://youtu.be/k3CleGTkHxE, Video: video showing the UAV-based system for on-site antenna measurement under operation.
